# COVID-19 vaccination, all-cause mortality, and hospitalization for cancer: 30-month cohort study in an Italian province

**DOI:** 10.17179/excli2025-8400

**Published:** 2025-07-01

**Authors:** Cecilia Acuti Martellucci, Angelo Capodici, Graziella Soldato, Matteo Fiore, Enrico Zauli, Roberto Carota, Marco De Benedictis, Graziano Di Marco, Rossano Di Luzio, Maria Elena Flacco, Lamberto Manzoli

**Affiliations:** 1 Department of Medical and Surgical Sciences, University of Bologna, 40100 Bologna, Italy; 2 Local Health Unit of Pescara, 65124 Pescara, Italy; 3 Department of Translational Medicine, University of Ferrara, 44121 Ferrara, Italy; 4 Department of Environmental and Prevention Sciences, University of Ferrara, 44121 Ferrara, Italy

**Keywords:** SARS-CoV-2, vaccines, all-cause mortality, cancer hospitalization, COVID-19

## Abstract

Anecdotal reports suggested an association between SARS-CoV-2 vaccination and some cancers, but no formal assessment has been published. This population-wide cohort analysis was aimed at evaluating the risk of all-cause death and cancer hospitalization by SARS-CoV-2 immunization status. Using National Health System official data, the entire population of the Pescara province, Italy was followed from June 2021 (six months after the first vaccination) to December 2023. Cox models were adjusted for age, gender, previous SARS-CoV-2 infection, and selected comorbidities. Of the 296,015 residents aged ≥11 years, 16.6% were unvaccinated, 83.3% received ≥1 dose, and 62.2% ≥3 doses. Compared with the unvaccinated, those receiving ≥1 dose showed a significantly lower likelihood of all-cause death, and a slightly higher likelihood of hospitalization for cancer (HR: 1.23; 95% CI: 1.11-1.37). The latter association was significant only among the subjects with no previous SARS-CoV-2 infection, and was reversed when the minimum time between vaccination and cancer hospitalization was set to 12 months. The subjects who received SARS-CoV-2 vaccination showed a substantial reduction in all-cause mortality, and a risk of cancer hospitalization that varied by infection status, cancer site, and the minimum lag-time after vaccination. Given that it was not possible to quantify the potential impact of the healthy vaccinee bias and unmeasured confounders, these findings are inevitably preliminary.

See also the graphical abstract(Fig. 1).

## Introduction

The vaccines against SARS-CoV-2 were authorized owing to the satisfactory efficacy-safety balance reported in clinical trials (Cheng et al., 2021[11]). Their effectiveness against severe disease and death due to COVID-19 was confirmed in further observational studies (Rosso et al., 2023[87]; Wu et al., 2023[103]). Subsequently, rare short-term and mid-term adverse events were detected in a number of countries by post-marketing surveillance (Choi et al., 2024[12]; Copland et al., 2024[16]; Dorajoo et al., 2023[18]; Faksova et al., 2024[22]; Mahasing et al., 2023[60]), and by more observational studies (Boker et al., 2024[8]; Fan et al., 2023[23]; Kumar et al., 2023[51]; Tsang et al., 2023[98]; Walton et al., 2023[100]; Yoon et al., 2023[106]). 

Since the early phases of vaccine roll-out, considerable efforts were made in order to implement passive surveillance systems to detect safety signals, and vaccine safety data-linking to verify such potential signals (Kesselheim et al., 2021[49]; Lo Re et al., 2021[59]; Rizzato Lede et al., 2022[85]). While, as mentioned, the short- and mid-term adverse events potentially related to vaccination were investigated in many studies, to this date evaluations are severely lacking about the theoretical long-term consequences of these vaccines (Seneff et al., 2022[93]). Indeed, some reports have hypothesized the potential of an oncogenic risk, given the novel nature of the majority of the distributed vaccines (Fendler et al., 2022[27]; McKernan et al., 2023[64]; Valdes Angues and Perea Bustos, 2023[99]; Wigner-Jeziorska et al., 2023[102]). The present cohort study evaluated the potential association between the anti-SARS-CoV-2 vaccines and the incidence of cancer hospitalization in the whole population of one Italian Province.

## Materials and Methods

This cohort study followed previous evaluations of vaccine effectiveness (Flacco et al., 2021[31]; Rosso et al., 2023[87]), and of potentially vaccine-related adverse events (Flacco et al., 2022[30]), and it included the population aged 11 years or older residing in the province of Pescara, Italy, on January 1st 2021. The aim was to compare the overall mortality and incidence of cancer in the vaccinated vs. the unvaccinated.

Vaccinated individuals were categorized in the following two groups: (1) persons who received one or more doses of Pfizer-BioNTech vaccine (BNT162b2), Moderna's mRNA vaccine (mRNA-1273), Oxford-AstraZeneca COVID-19 vaccine (ChAdOx1 nCoV-19), or Novavax COVID-19 vaccine (NVX-CoV2373) (included in the group "≥1 dose"); (2) individuals who received three or more doses of any of the above COVID-19 vaccines, or two or more vaccine doses, if one of the administered vaccines was Johnson & Johnson COVID-19 vaccine (JNJ-78436735) (included in the group "≥3 doses").

### Data collection

We extracted the following datasets, that are routinely compiled and entered into the Italian National Health System official database of the Pescara Local Health Unit: COVID-19 (swabs), demographic, SARS-CoV-2 vaccination, hospital admissions (Italian “SDO”), and co-pay exemption (“Esenzioni Ticket” file). The encrypted fiscal codes were used to perform deterministic linkage of all datasets, which include information on all the residents of the Pescara province.

### Outcomes 

The main outcomes were (a) the rate of first hospital admissions for cancers of any site (with the exclusion of skin cancers), and (b) all-cause mortality. We also separately evaluated the rates of first hospitalizations for the six cancers that were most frequently diagnosed in Italy in 2023 (AIRTUM and AIOM, 2023[3]), and the rates of first hospitalizations for three additional cancers based upon the reported bio-distribution of the vaccine-induced spike protein (European Medicines Agency, 2021[21]; Pateev et al., 2023[80]). 

The hospital admissions for cancer were identified using the following International Classification of Diseases, Ninth Revision, Clinical Modification (ICD9-CM) codes in any diagnosis field: 140.XX to 172.XX, and 174.XX to 209.XX (all cancers); 162.XX (lung); 153.XX to 154.XX (colorectal), 174.XX (breast), 185.XX (prostate), 182.XX (uterine body), 188.XX (bladder), 193.XX (thyroid), 183.XX (ovarian), 186.XX (testicular). Only the subjects who were admitted for the above cancers for the first time after the follow-up start were considered as new cases. As an example, if a person had one or more hospital admission for prostate cancer in the last ten years before the start of the follow-up, he was excluded from the analyses on the risk of hospitalization for prostate cancer.

### Follow-up

Due to the uncertain timing of the potential oncogenic effect after vaccination, a putative period of 180 days was chosen as the minimum time between exposure and possible outcome. 

For the unvaccinated, the follow-up started (a) on June 27, 2021 (180 days after the start of the immunization campaign, on January 1, 2021) for the comparison between unvaccinated and the group "≥1 dose"; (b) on December 26, 2021 (180 days after the first administration of the third dose, on July 1, 2021) for the comparison between the unvaccinated individuals and the subjects who received ≥3 doses. 

For the vaccinated individuals, the follow-up started (a) 180 days after the first dose for all the individuals who received ≥1 dose; (b) 180 days after the third dose (or the second dose for recipients of the JNJ-78436735 vaccine) for the individuals who received ≥3 doses. 

For both the exposed and the non exposed, the follow-up ended the day of the first admission for the subjects who had a cancer admission, or the day of the death, or on December 31, 2023 for those without a cancer admission. 

### Statistical analyses

Cox proportional hazards models were fit to explore the potential association between the considered outcomes and exposures, calculating hazard ratios (HRs) and their 95% confidence intervals (CIs). All multivariable models were adjusted a priori for the following covariates: age (both categorical and as a quadratic term), gender, previous SARS-CoV-2 infection, and selected comorbidities identified through the following ICD9-CM codes in any diagnosis field: 250.xx (diabetes); 401.xx-405.xx (hypertension); 410.xx-412.xx, 414.xx-415.xx, 428.xx, or 433.xx-436.xx (CVD); 491.xx-493.xx (COPD); 580.xx-589.xx (kidney disease); and 140.xx-172.xx or 174.xx-208.xx (admission for cancer prior to the start of the follow-up). Previous SARS-CoV-2 infections were only considered if they occurred more than 180 days before the end of follow-up, to allow enough time for a potential independent modifying effect on the investigated outcomes.

A minimum events-to-variable ratio of 10 was maintained in all models, while the validity of proportional hazard assumptions and of constant incidence ratios up to follow-up were tested using respectively Schoenfeld's test and Nelson-Aalen cumulative hazard estimates (Hosmer et al., 1999[40]). The significance level was set as a p-value < 0.05, and all analyses were performed using Stata, version 13.1 (Stata Corporation, College Station, TX, USA, 2014).

As sensitivity analyses, given the uncertain timing of the potential oncogenic effect after vaccination, we repeated all analyses adopting two different starts of follow-up: (1) adding a minimum period of 90 days, instead of 180, from the start of the vaccination campaign (or the first or third vaccine dose) and the possible outcome; (2) adding a minimum period of 365 days, instead of 180, from the start of the vaccination campaign (or the first or third vaccine dose) and the possible outcome. As with the main analyses, in people with an outcome, previous SARS-CoV-2 infections were only considered if they occurred more than 90 days before the end of follow-up.

## Results

The analysis included all 296,015 residents or domiciled individuals in the province of Pescara, Italy, from the beginning of the vaccination campaign (January 1, 2021) to December 31, 2023, after excluding 28,267 subjects who were 10 years of age or younger, 2298 hospitalizations of non-residents or non-domiciled individuals, and 171 incorrect fiscal codes.

### Sample characteristics

Of the 296,015 overall population, 48.9% were males, 16.6% were unvaccinated (n=49,265), 83.3% were vaccinated with at least one dose (n=246,750), and 62.2% received at least three doses (n=183,999 - Table 1(Tab. 1)). Almost half (49.7%) of the subjects who received at least three doses received a mixed schedule, 38.0% received BNT162b2, and 11.8% received mRNA-1273.

A markedly younger age was observed among the unvaccinated (mean 45.1±19.7 years), compared to the subjects who received at least three vaccine dose (mean 53.0±20.0 years; p<0.001; Student's t-test for unpaired samples) or at least one dose (mean 50.2±20.5 years; p<0.001). This was consistent with the higher rates of comorbidities and previous hospitalizations for any cancer that was observed among the vaccinated individuals. A previous SARS-CoV-2 infection was recorded in 29.3% of the unvaccinated, 43.5% of those who received at least one vaccine dose, and 37.2% of those who received at least three doses. Finally, the mean follow up was 29.3 months for the unvaccinated, 23.9 months for the group "≥1 dose", and 17.6 months for the group "≥3 doses".

### All-cause mortality

Overall, 6512 subjects died during the follow-up (2.20% of the sample; Table 2(Tab. 2)). The mortality among the unvaccinated (3.56%) was much higher than among those who received at least one dose (1.93%; p<0.001; chi-squared test) or at least three vaccine doses (1.30% vs. 2.07% in the unvaccinated; p<0.001). Multivariate analyses confirmed univariate results, showing a significantly lower risk of death for the group "≥1 dose" (HR: 0.42; 95% CI 0.39-0.44) and for the group "≥3 doses" (HR: 0.65; 0.62-0.67; Table 3(Tab. 3)), as compared with the unvaccinated. Similar results were observed in all stratified (Tables S1 and S2) and sensitivity analyses (Tables S3 and S4).

### All cancer hospitalizations

Overall, 3134 subjects had a hospital admission with a cancer diagnosis during the follow-up (1.10% of the sample; Table 2(Tab. 2)). The rate of hospitalization for cancer of any site was 0.85% in the unvaccinated group, and 1.15% in the group vaccinated with at least one dose (p<0.001). At multivariate analyses, the likelihood of cancer hospitalization was higher in the subjects who received at least one dose, compared to the unvaccinated (HR: 1.23; 1.11-1.37; Table 3(Tab. 3)). Similar results were observed for the vaccinated with at least three doses (HR: 1.09; 1.02-1.16). 

When the analyses were stratified by gender, a higher risk of cancer hospitalization was seen only among males vaccinated with at least one dose (HR: 1.31; 1.12-1.52; Table S1). Instead, after stratifying by previous SARS-CoV-2 infections, cancer hospitalization was more likely among individuals without a reported previous infection, whether vaccinated with one or more doses (HR: 1.31; 1.16-1.47) or with three or more doses (HR: 1.11; 1.03-1.20). Finally, after stratifying by vaccine type, all except mRNA-1273 were positively associated with the overall cancer hospital admissions (Table S2). The sensitivity analyses using at least 90 days, instead of 180, between the start of the vaccination campaign (or the first or third vaccine dose) and the first cancer hospitalization (Table S3) showed no substantial differences. Instead, in the sensitivity analyses using at least 365 days, the association of cancer risk with ≥1 vaccine dose was not significant anymore, whereas the individuals who received ≥3 doses showed a significantly lower risk of hospitalization (HR 0.90; 0.83-0.98; Table S4).

### Cancer hospitalizations by site

At univariate analyses, the vaccinated subjects showed higher rates of cancer admission for colon-rectum, breast, bladder, and all hematological cancers (the latter only for the comparison with the group "≥1 dose"; Table 2(Tab. 2)). The multivariate models largely confirmed the univariate analyses. Vaccination with at least one dose was significantly associated with a higher risk of hospitalization for colon-rectum cancer (HR: 1.34; 1.00-1.80), breast cancer (HR: 1.54; 1.10-2.16), and bladder cancer (HR: 1.62; 1.07-2.45; Table 3(Tab. 3)). After three or more vaccine doses, similar results were observed for breast cancer (HR: 1.36; 1.08-1.72), and for bladder cancer (HR: 1.43; 1.08-1.88).

While the higher risk of bladder cancer was observed only among the males (Table S1), contrasting results were observed when the analyses were stratified by previous SARS-CoV-2 infection. Among people without a previous infection, a positive and significant association was observed between vaccination and hospitalizations for cancers of four sites. Conversely, among those with a previous infection, this association was either absent or negative.

**Figure 2:**
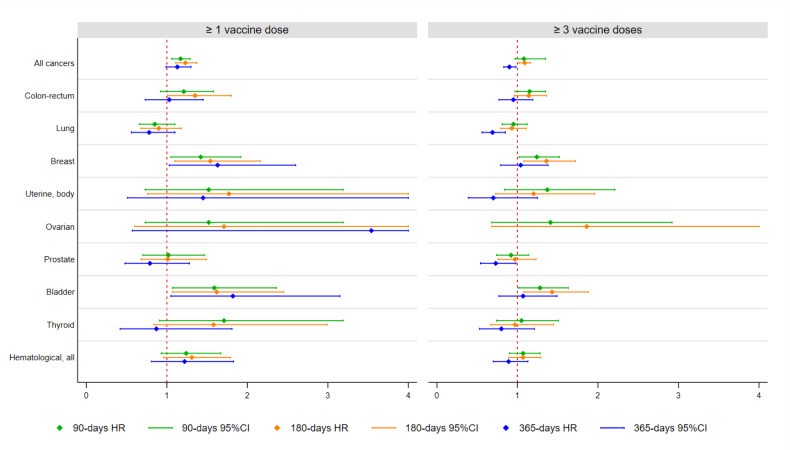
Adjusted hazards ratios (HR) and 95% confidence intervals (CI) ^A^ of hospitalization for all cancers and selected cancers, by exposure to ≥ 1 or ≥ 3 vaccine doses ^B^ and by lag-time. The unvaccinated group is the reference category for all analyses. ^A^ Based on Cox proportional hazards models, adjusted for gender, age, diabetes, hypertension, cardiovascular or cerebrovascular disease, chronic obstructive pulmonary disease, kidney disease, and infection status. ^B ^When comparing the unvaccinated versus the subjects who received at least one dose, the follow-up started on March 30, 2021 for the unvaccinated individuals, and after the selected lag-times from the first dose for the vaccinated subjects. When comparing the unvaccinated versus the subjects who received at least three doses, the follow-up started on September 28, 2021 for the unvaccinated, and after the selected lag-times from the third dose for the vaccinated subjects.

Furthermore, the risk of hospitalization was increased for cancers of the breast and the bladder with any type of vaccine, for hematological cancers with BNT162b2 or ChAdOx1 nCoV-19, and finally for colonrectum cancers with a mixed schedule (Table S2). No significant associations were found between vaccination and hospitalization for the neoplasms of the lung, ovaries, and thyroid, and no substantial discrepancies were detected at sensitivity analyses with a minimum time of 90 days set between vaccination and the first hospitalization (Table S3). Importantly, when a minimum time of 365 days was set, while breast and bladder cancer hospitalizations maintained their positive association with ≥1 dose, the individuals who received ≥3 doses showed a significantly lower likelihood of hospitalization for lung or prostate cancer (Table S4). The results of the main multivariable analyses predicting cancer hospitalization at 90, 180 and 365 days have been summarized in Figure 2(Fig. 2).

## Discussion

In this cohort study, which followed all the residents of an Italian province for up to 30 months, SARS-CoV-2 vaccination showed a strong, negative association with all-cause mortality, while the likelihood of cancer hospitalization of the vaccinated individuals varied substantially, depending on infection status, cancer site, and the minimum lag-time between vaccination and cancer.

While evidence is abundant on the vaccination effectiveness against COVID-19 deaths (Acuti Martellucci et al., 2022[1]; Flacco et al., 2021[31]; Rosso et al., 2023[87]; Wu et al., 2023[103]), an increasing number of studies reported a high impact against all-cause and non-COVID-19 mortality (though waning with time), worldwide (Horne et al., 2022[39]; B. Liu et al., 2023[57]; Pálinkás and Sándor, 2022[76]; Xu et al., 2023[104]) and in the same province (Flacco et al., 2022[30]; Rosso et al., 2023[87]). Clearly, the 40% risk reduction in all-cause mortality observed in our study, exceeds the impact that could be expected from the reduction of COVID-19 related mortality, which was estimated to cause less than 30% of the excess mortality registered in a number of countries (Bielinski et al., 2024[6]; Mostert et al., 2024[69]; Wang et al., 2022[101]). However, as previously reported for these and other vaccines (Chung et al., 2021[13]; Flacco et al., 2022[30]), this discrepancy was likely caused by the healthy vaccine bias (Høeg et al., 2023[38]), as the vaccinated individuals are well known to be more likely, as compared with the unvaccinated ones, to present further unmeasured characteristics which might protect them from death (Remschmidt et al., 2015[84]).

As regards the observed association between SARS-CoV-2 vaccination and cancer incidence rates, both positive and negative, besides anecdotal reports (Eens et al., 2023[20]; Goldman et al., 2021[35]; Kyriakopoulos et al., 2023[53]; Mizutani et al., 2022[68]; Olszewska et al., 2024[75]; Zamfir et al., 2022[107]) no published study has previously evaluated the potential association between cancer risk and vaccination status, and only one study investigated the possible impact of COVID-19 vaccines on cancer mortality (Fedeli et al., 2024[25]). This analysis found higher mortality rates for cancer in 2021 and 2022 compared to 2020 in the U.S. (Fedeli et al., 2024[25]). However, this study did not directly compare vaccinated vs. unvaccinated subjects, and the increases in cancer mortality could clearly be due to a direct effect of the SARS-CoV-2 infection, as well as to the delays in the cancer diagnostic systems observed during the pandemic (Muka et al., 2023[70]). In the present study, while diagnostic delays and further confounders cannot be excluded, it should also be mentioned that the healthy vaccinee bias, similarly to how it likely leads to and overestimation of vaccine effectiveness against all-cause death, could also lead to an underestimation of the potential negative impact of vaccination on hospitalization due to cancer. Indeed, the healthier lifestyle that is typically associated with vaccination may reduce the risk of lifestyle-associated carcinomas.

Aiming to verify the potential effect of both vaccination and natural infection (Jahankhani et al., 2023[46]; Roncati et al., 2023[86]), infection status was used to adjust all the multivariate models, together with age, gender, and selected comorbidities. When the analyses were stratified by infection status, the results were sharply different among the infected and the uninfected: in the analyses restricted to the people without a certified SARS-CoV-2 infection (recorded at least six months before the cancer diagnosis), vaccinated subjects showed a small, significant increase in new cancer hospitalizations. In contrast, no association between vaccines and cancer was observed among the individuals with a recorded previous infection. Even if we could not exclude a potential role of the vaccines over and beyond SARS-CoV-2 infection, such sharp differences by infection status should be interpreted with caution: while it is possible that seropositivity may modulate the response to vaccination (Chambers et al., 2024[9]; Leung, 2022[56]), or that the infection itself may modulate the immune response to cancer cells (Xianpeng Liu et al., 2024[58]), it is also true that in the study setting the requirements for SARS-CoV-2 testing and vaccination changed frequently (Italian Government, 2022[44]), and an unknown portion of those resulting uninfected were likely not tested (Flacco et al., 2022[32]). 

The theoretical arguments supporting a potential tumorigenic action of the mRNA anti-SARS-CoV-2 vaccines have been reported in four reviews, which gathered evidence from studies on the biological responses to vaccination in animals and humans (Igyártó and Qin, 2024[42]; Polykretis et al., 2023[82]; Seneff et al., 2022[93]; Valdes Angues and Perea Bustos, 2023[99]). According to these authors, vaccination may promote or expedite the oncogenic multi-hit process through the following mechanisms: (1) pro-inflammatory and tumorigenic effects triggered by the vaccine mRNA and vaccine-induced Spike protein, both systemically and on mucosal surfaces such as the gut (Kobbe et al., 2024[50]; Nascimento et al., 2024[71]; Parry et al., 2023[79]; Rubio-Casillas et al., 2024[89]; Zeng et al., 2022[108]); (2) pro-inflammatory action of LNPs, whose biodistribution was reported for almost every organ (Bahl et al., 2017[4]; Fertig et al., 2022[29]; Hanna et al., 2023[37]; Maruggi et al., 2022[62]; Ng et al., 2022[73]; Pateev et al., 2023[80]); (3) altered translation of cellular microRNA; (4) reduced Interferon type 1 activity (Franco et al., 2023[33]); and finally (5) lymphopenia, also observed by Sing et al. (Sing et al., 2022[95]), possibly related to an untimely cytokine signal which inactivates T-cells (Igyártó and Qin, 2024[42]). Notably, the lymphopenia and the pro-inflammatory action of mRNA were also reported for SARS-CoV-2 infection (Valdes Angues and Perea Bustos, 2023[99]).

In fact, a number of publications reported various manifestations related to a deregulation of the immune system following vaccination against SARS-CoV-2 (Chen et al., 2022[10]; Cinicola et al., 2022[14]; Federico, 2024[26]; Jung et al., 2024[48]; Sacchi et al., 2023[91]). For instance, the mRNA vaccines were found to be associated with immune-mediated adverse events such as Herpes Zoster flares (Fathy et al., 2022[24]; Nelli et al., 2024[72]), immune thrombocytopenia and other autoimmune hematological disorders (Barda et al., 2021[5]; Mingot-Castellano et al., 2022[67]), and also neurological, otolaryngology, renal, skin, ocular, and thyroid manifestations (Ahmed et al., 2022[2]; Colizza et al., 2022[15]; Habot-Wilner et al., 2023[36]; Hosseini and Askari, 2023[41]; Kuziez et al., 2023[52]; McMahon et al., 2021[65]; Meo et al., 2024[66]; Şendur et al., 2023[92]; Zhang et al., 2022[109]). Additionally, the Adenovirus vector vaccine was found to induce thrombocytopenia, thrombosis, and capillary leak syndrome (Dabbiru et al., 2023[17]; Faksova et al., 2024[22]; Ruggiero et al., 2022[90]). Lastly, a final hypothesis in need of validation has been proposed, to date, by two preprint papers (McKernan et al., 2023[64]; Speicher et al., 2023[96]). Namely, the BNT162b2 and mRNA-1273 vaccines could be contaminated with detectable quantities of DNA, which are likely to promote oncogenesis (Rotondo et al., 2019[88]). As mentioned initially, all these theories need validation, and, combined with the contrasting results of the present cohort study, strongly call for further investigation, providing indications for future research directions.

Concerning the observed differences in cancer location, research on the potential impact of vaccination at the organ-level is in its early stages. According to the scarce available evidence, vaccine mRNA was detected in human breast milk (Hanna et al., 2023[37]; Yeo et al., 2022[105]), alterations were observed in the urinary proteome and urologic immunity (Pan et al., 2022[77]; Shim et al., 2023[94]), and a potential role was hypothesized in the pathogenesis of hematological malignancies (Gentilini et al., 2024[34]; Olszewska et al., 2024[75]). Interestingly, gender did not seem to influence either cellular or humoral responses to anti-SARS-CoV-2 vaccines (Chambers et al., 2024[9]), therefore the gender difference in the risk of cancer hospitalization will have to be explored in future studies.

As with all-cause mortality, it is possible that unmeasured variables affected the results on cancer hospitalization: in particular, since the vaccinees could be more prone than non-vaccinees to seek healthcare (McElfish et al., 2023[63]; Oancea and Watson, 2019[74]), they could also be more likely to receive a cancer diagnosis, which may explain the higher hospitalization rate observed in some of the analyses. Moreover, the SARS-CoV-2 pandemic was characterized by a surge of mistrust in the healthcare systems (Biswas et al., 2021[7]), which could have potentially impacted both the probability of being vaccinated and being hospitalized for cancer (Fenta et al., 2023[28]), and could contribute to explain the findings of a positive association between COVID-19 vaccination and cancer hospitalization. This also fits with another result of the study: the hazard of being hospitalized for cancer was higher in individuals that received at least one vaccine dose, compared to the unvaccinated, but did not increase when the analyses were restricted to those exposed to at least three doses. Such an apparent lack of dose-response could either challenge the hypothesis of oncogenesis, suggesting that the observed associations are to be attributed to unmeasured, confounding factors, or just indicate that a single dose could be sufficient to trigger the potential tumorigenic action.

Overall, as this is the first study to report a significantly higher risk of cancer hospitalization after anti-SARS-CoV-2 vaccination in some of the analyses, all the hypotheses about the biological plausibility and the potential explanations of such an association must be considered provisional. 

### Strengths and limitations

This study examined the whole population of one Italian province, amounting to almost 300,000 individuals, and used official healthcare datasets to record all hospitalizations, vaccines, swabs, and demographics from the inception of the vaccination campaign, for a maximum follow-up of 30 months. However, the study also has important limitations which should be mentioned. First, as discussed above, similarly to all observational studies, residual confounding cannot be excluded. Second, although hospital discharge abstract, SDOs, represent one of the main sources of data to estimate the incidence of cancer diagnosis in Italian cancer registries (International Agency for Research on Cancer, 2024[43]; Tognazzo et al., 2014[97]), when used alone they represent only a proxy of the total new cases of cancer. However, Italian cancer registries are currently processing data with an average delay of 3-5 years, with individual data for the year 2024 available no sooner than in 2028, motivating the use of hospital discharge abstracts alone, though suboptimal, to estimate cancer diagnoses in Italy (Parazzini et al., 2017[78]; Piscitelli et al., 2009[81]) and other countries (Ji et al., 2012[47]; Lee et al., 2022[55]; Porter et al., 1984[83]). Third, as the smoking status was unknown, the association between vaccination and cancer incidence could be overestimated in the event that vaccine uptake was positively associated with smoking. Unfortunately, the Italian National Health System does not routinely collect data on smoking status and other potential confounders (e.g. healthcare literacy, which would allow an assessment of the healthy vaccinee bias). However, compared to non-smokers, smokers were reported to be either less or equally likely to get vaccinated against SARS-CoV-2 (Ebrahimi Kalan et al., 2023[19]; Jackson et al., 2021[45]; Lastrucci et al., 2022[54]). Fourth, while all the deaths were captured, the lack of pathology data could have led to missing some cancers, as some individuals with early-stage neoplasms may not necessarily need hospitalization. As the present evaluation did not detect these pathology-diagnosed cancers, the observed positive association between anti-SARS-CoV-2 vaccines and cancer hospitalizations may result from a higher vaccination uptake among hospitalized patients, and further data are required to verify this hypothesis.

Ideally, future studies should evaluate the potential association between vaccination and cancer incidence through linkage analyses of SARS-CoV-2 vaccination data, cancer incidence data from cancer registries, and information on potential confounders from general practitioners. These data sources, on a sufficiently large population, permit to capture all cancer diagnoses (from both hospital admissions and pathology tests), and adjust for lifestyle behaviors (provided by general practitioners). In Italy, however, cancer registry data are typically available with the long delay mentioned above, and GPs' datasets often lack basic information (Manzoli et al., 2010[61]), and require specific, expensive agreements to be accessed.

## Conclusions

The subjects who received SARS-CoV-2 vaccination showed almost half the risk of all-cause death after a median follow-up of 25 months. We also observed an inconstant association between COVID-19 vaccination and cancer hospitalization, depending on infection status, cancer site, and the minimum lag-time between vaccination and cancer. As the results might be influenced by the confounding effect of a differential healthcare utilization by vaccinated individuals, they must be considered preliminary, and further data are definitely required to elucidate the potential association between cancer and COVID-19 vaccination.

## Notes

Cecilia Acuti Martellucci and Angelo Capodici contributed equally to this work.

## Declaration

### Supplementary materials

Table S1: Adjusted hazards ratios (95% confidence interval ‒ CI) of all-cause death, all cancers, and selected cancers, stratified by gender and infection status. The unvaccinated group is the reference category for all analyses. Table S2: Adjusted hazards ratios (95% confidence interval ‒ CI) of all-cause death, all cancers, and selected cancers, stratified by type of vaccine. The unvaccinated group is the reference category for all analyses. Table S3: Adjusted hazards ratios (95% confidence interval ‒ CI) of all cancers, and selected cancers. The unvaccinated group is the reference category for all analyses. Sensitivity analyses adopting a different start of follow-up: adding a minimum period of 90 days, instead of 180, from the start of the vaccination campaign (or the first or third vaccine dose) and the possible outcome. Table S4: Adjusted hazards ratios (95% confidence interval ‒ CI) of all cancers, and selected cancers. The unvaccinated group is the reference category for all analyses. Sensitivity analyses adopting a different start of follow-up: adding a minimum period of 395 days, instead of 180, from the start of the vaccination campaign (or the first or third vaccine dose) and the possible outcome.

### Author contributions

Conceptualization, C.A.M., M.E.F. and L.M.; methodology, A.C., E.Z., M.F. and C.A.M.; software, R.C., M.D.B., and G.D.M.; validation, M.E.F., L.M. and R.D.L.; formal analysis, M.D.B. and G.D.M.; investigation, A.C., E.Z., M.F. and R.C.; resources, G.S. and R.D.L.; data curation, R.C., M.D.B. and G.D.M.; writing ‒ original draft preparation, C.A.M., A.C., E.Z. and M.F.; writing ‒ review and editing, L.M. and M.E.F.; supervision, G.S. and L.M.; project administration, G.S., R.D.L. and M.E.F. All authors have read and agreed to the published version of the manuscript.

### Artificial Intelligence (AI) - Assisted Technology

None was used in any stage of this work.

### Funding

This research received no external funding.

### Institutional Review Board Statement

The study was conducted in accordance with the Declaration of Helsinki, and approved by the Ethics Committee of the Emilia-Romagna Region (protocol code 287, approved on 24 March 2020).

### Informed Consent Statement

Patient consent was waived due to the retrospective and pseudo-anonymized nature of the data. According to the European Union General Data Protection (GDPR) regulation, all datasets were pseudo-anonymized (using a unique identification code for each subject in each dataset) and analyzed by the NHS Offices before access of the authors. All data concerning the address, phone number, email, date of birth, vaccination center, hospital site, swab lab, and municipality of all subjects were not provided to the authors, and the encrypted identification code could not be reversed by the regional offices (the encryption was made in two steps by assigning random codes for each fiscal code in the demographic database, and the intermediate codes were deleted). R.C. and M.D.B. performed the data processing and have permission to release anonymized raw data upon request.

### Data Availability Statement

The data used for this study are available from the corresponding author upon reasonable request.

### Conflict of Interest

The authors declare no conflicts of interest.

## Supplementary Material

Supplementary information

## Figures and Tables

**Table 1:**
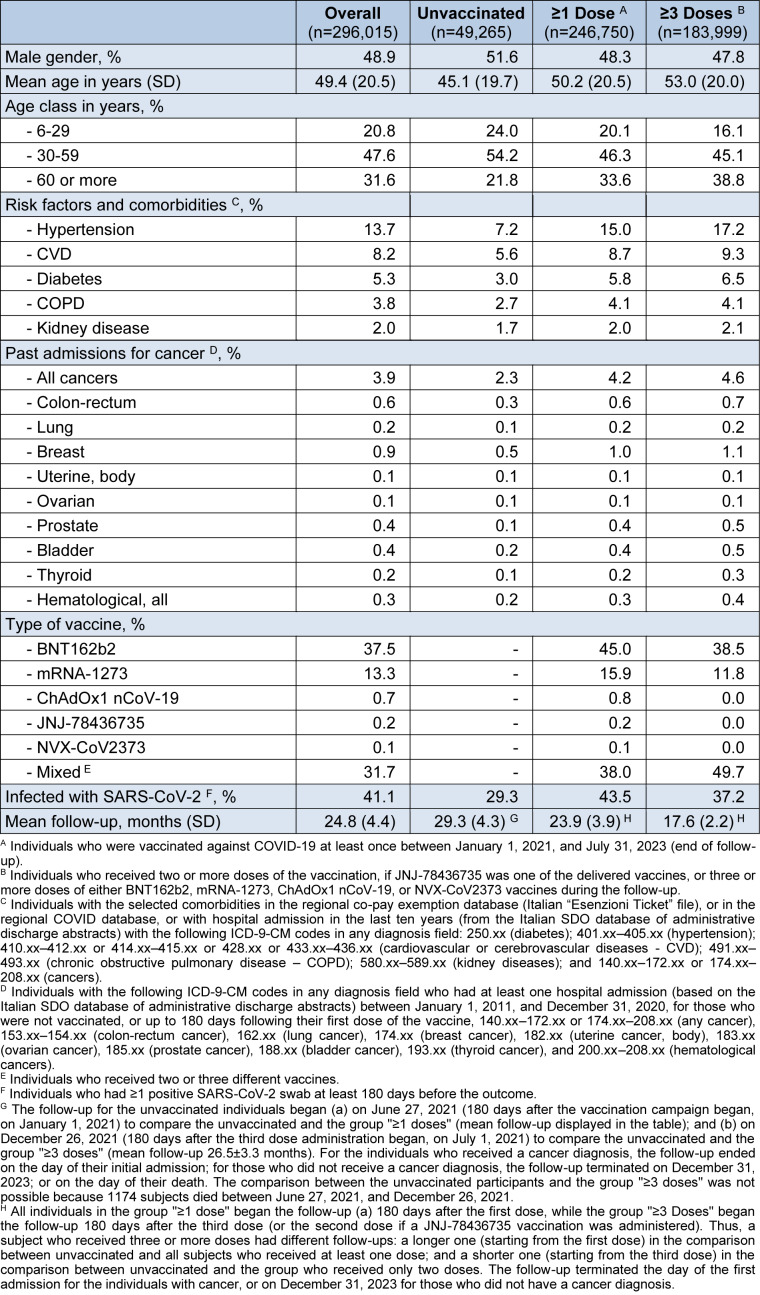
Characteristics of the sample, overall and by COVID-19 vaccine status.

**Table 2:**
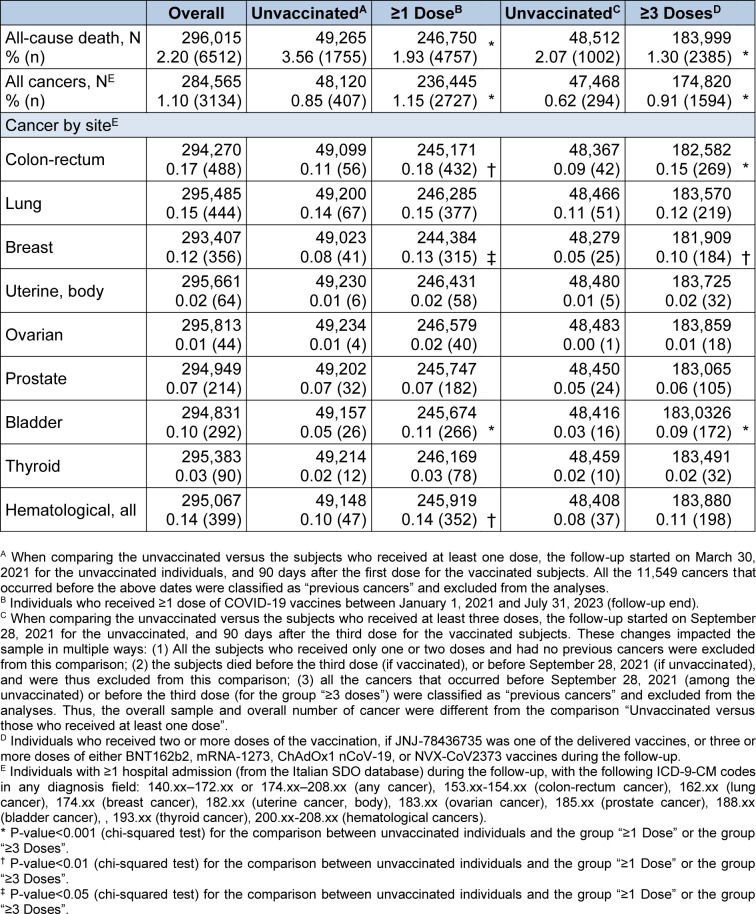
Main outcomes, overall and by COVID-19 vaccine status.

**Table 3:**
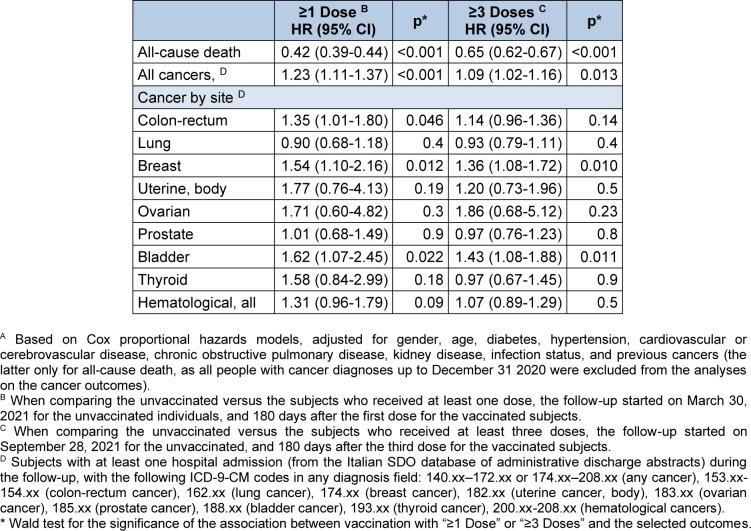
Adjusted hazards ratios (95% confidence interval ‒ CI) ^A^ of all-cause death, all cancers, and selected cancers. The unvaccinated group is the reference category for all analyses.

**Figure 1:**
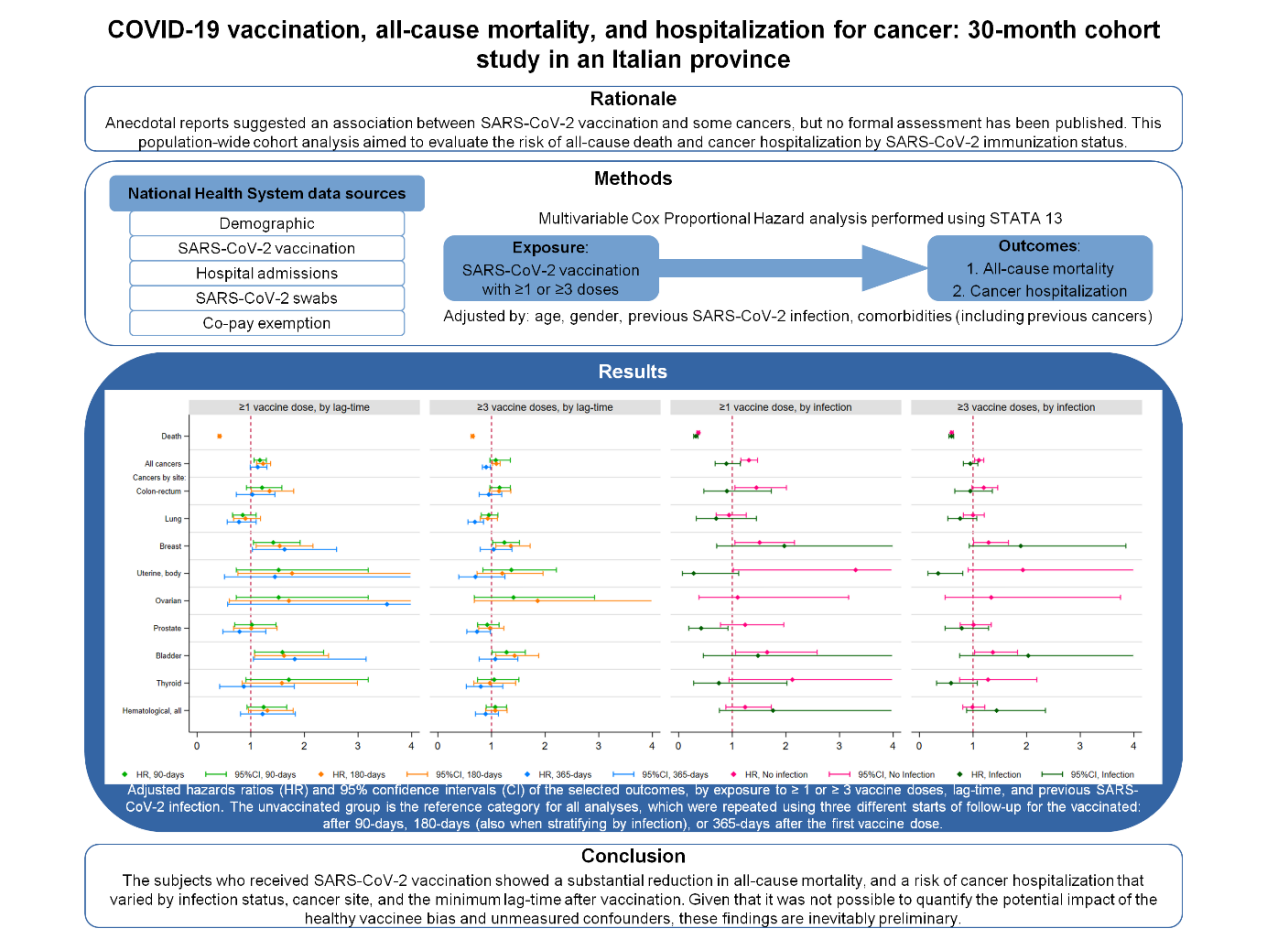
Graphical abstract
