# Supplementary material for: COVID-19 vaccination, all-cause mortality, and hospitalization for cancer: 30-month cohort study in an Italian province
Source: EXCLI J. 2025 Jul 1;24:690–707. doi: 10.17179/excli2025-8400 (PMC12381369; doi:10.17179/excli2025-8400)
Supplement: Supplementary information [file EXCLI-24-690-s-001.pdf]

EXCLI Journal 2025;24:690-707 – ISSN 1611-2156
Received: March 30, 2025, accepted: May 07, 2025, published: July 01, 2025

Supplementary data to:

Original article:

COVID-19 VACCINATION, ALL-CAUSE MORTALITY, AND HOSPITALIZATION FOR CANCER: 30-MONTH COHORT STUDY IN AN ITALIAN PROVINCE

Cecilia Acuti Martellucci[1,#], Angelo Capodici[1,#], Graziella Soldato[2], Matteo Fiore[1], Enrico Zauli[3], Roberto Carota[2], Marco De Benedictis[2], Graziano Di Marco[2], Rossano Di Luzio[2], Maria Elena Flacco[4], Lamberto Manzoli[1,*]

[1] Department of Medical and Surgical Sciences, University of Bologna, 40100 Bologna, Italy; c.acutimartellucci@unibo.it (C.A.M.); angelo.capodici@studio.unibo.it (A.C.); matteo.fiore7@studio.unibo.it (M.F.); lamberto.manzoli2@unibo.it (L.M.)
[2] Local Health Unit of Pescara, 65124 Pescara, Italy; graziella.soldato@ausl.pe.it (G.S.); roberto.carota@ausl.pe.it (R.C.); marco.debenedictis@ausl.pe.it (M.D.B.); graziano.dimarco@ausl.pe.it (G.D.M.); rossano.diluzio@ausl.pe.it (R.D.L.)
[3] Department of Translational Medicine, University of Ferrara, 44121 Ferrara, Italy; enricozauli8@gmail.com (E.Z.)
[4] Department of Environmental and Prevention Sciences, University of Ferrara, 44121 Ferrara, Italy; mariaelena.flacco@unife.it (M.E.F.)

# These authors contributed equally to this work.

* **Corresponding author:** Lamberto Manzoli, Department of Medical and Surgical Sciences, University of Bologna, 40100 Bologna, Italy.
Email: lmanzoli@post.harvard.edu, Tel.: +39 3474727282

https://dx.doi.org/10.17179/excli2025-8400

This is an Open Access article distributed under the terms of the Creative Commons Attribution License (https://creativecommons.org/licenses/by/4.0/).

EXCLI Journal 2025;24:690-707 – ISSN 1611-2156
Received: March 30, 2025, accepted: May 07, 2025, published: July 01, 2025

Table S1: Adjusted hazards ratios (95% confidence interval – CI) A of all-cause death, all cancers, and selected cancers, stratified by gender and infection status. The unvaccinated group is the reference category for all analyses.

	Male		Female		No previous SARS-CoV-2 infection		Previous SARS-CoV-2 infection	
	≥1 Dose [B] HR (95% CI)	≥3 Doses [C] HR (95% CI)	≥1 Dose [B] HR (95% CI)	≥3 Doses [C] HR (95% CI)	≥1 Dose [B] HR (95% CI)	≥3 Doses [C] HR (95% CI)	≥1 Dose [B] HR (95% CI)	≥3 Doses [C] HR (95% CI)
All-cause death	0.41 (0.38-0.45)	0.64 (0.61-0.68)	0.33 (0.30-0.35)	0.58 (0.55-0.61)	0.37 (0.34-0.39)	0.60 (0.58-0.63)	0.32 (0.28-0.36)	0.60 (0.55-0.64)
All cancers, [D]	1.31 (1.12-1.52)	1.07 (0.97-1.16)	1.14 (0.98-1.33)	1.09 (0.99-1.20)	1.31 (1.16-1.47)	1.11 (1.03-1.20)	0.89 (0.68-1.15)	0.95 (0.82-1.09)
Cancer by site [D]								
- Colon-rectum	1.42 (0.94-2.15)	1.14 (0.89-1.46)	1.24 (0.82-1.88)	1.13 (0.89-1.45)	1.45 (1.05-2.01)	1.20 (0.98-1.46)	0.90 (0.47-1.73)	0.95 (0.66-1.36)
- Lung	0.99 (0.69-1.42)	0.96 (0.77-1.19)	0.74 (0.49-1.13)	0.88 (0.67-1.15)	0.94 (0.70-1.26)	1.00 (0.82-1.21)	0.70 (0.33-1.45)	0.76 (0.53-1.07)
- Breast	-	-	1.54 (1.10-2.16)	1.36 (1.08-1.72)	1.51 (1.05-2.16)	1.29 (1.01-1.66)	1.97 (0.71-5.47)	1.89 (0.93-3.85)
- Uterine, body	-	-	1.76 (0.75-4.11)	1.19 (0.73-1.95)	3.30 (1.02-10.7)	1.93 (0.91-4.07)	0.28 (0.07-1.12)	0.35 (0.16-0.81)
- Ovarian	-	-	1.69 (0.60-4.77)	1.84 (0.67-5.05)	1.10 (0.38-3.17)	1.34 (0.48-3.75)	NE	NE
- Prostate	1.02 (0.69-1.52)	0.97 (0.76-1.24)	-	-	1.24 (0.78-1.96)	1.01 (0.76-1.34)	0.42 (0.19-0.92)	0.79 (0.48-1.29)
- Bladder	1.79 (1.10-2.92)	1.48 (1.07-2.06)	1.21 (0.55-2.66)	1.29 (0.77-2.17)	1.65 (1.06-2.58)	1.37 (1.03-1.83)	1.48 (0.46-4.81)	2.03 (0.75-5.52)
- Thyroid	1.67 (0.49-5.71)	0.67 (0.28-1.60)	1.46 (0.70-3.04)	1.04 (0.68-1.61)	2.12 (0.94-4.81)	1.28 (0.75-2.19)	0.75 (0.28-2.02)	0.59 (0.32-1.08)
- Hematological, all	1.22 (0.81-1.84)	0.98 (0.77-1.25)	1.44 (0.89-2.33)	1.20 (0.88-1.62)	1.24 (0.88-1.73)	0.99 (0.81-1.22)	1.76 (0.76-4.08)	1.44 (0.88-2.35)

[A] Based on Cox proportional hazards models, adjusted for age, gender, infection status, diabetes, hypertension, cardiovascular or cerebrovascular disease, chronic obstructive pulmonary disease, kidney disease, and previous cancers (the latter only for all-cause death, as all people with cancer diagnoses up to December 31 2020 were excluded from the analyses on the cancer outcomes).
[B] When comparing the unvaccinated versus the subjects who received at least one dose, the follow-up started on March 30, 2021 for the unvaccinated individuals, and 180 days after the first dose for the vaccinated subjects.
[C] When comparing the unvaccinated versus the subjects who received at least three doses, the follow-up started on September 28, 2021 for the unvaccinated, and 180 days after the third dose for the vaccinated subjects.
[D] Subjects with at least one hospital admission (from the Italian SDO database of administrative discharge abstracts) during the follow-up, with the following ICD-9-CM codes in any diagnosis field: 140.xx–172.xx or 174.xx–208.xx (any cancer), 153.xx-154.xx (colon-rectum cancer), 162.xx (lung cancer), 174.xx (breast cancer), 182.xx (uterine cancer, body), 183.xx (ovarian cancer), 185.xx (prostate cancer), 188.xx (bladder cancer), 193.xx (thyroid cancer), 200.xx-208.xx (hematological cancers).
NE = not estimable due to the scarcity of observations.

EXCLI Journal 2025;24:690-707 – ISSN 1611-2156
Received: March 30, 2025, accepted: May 07, 2025, published: July 01, 2025

Table S2: Adjusted hazards ratios (95% confidence interval – CI) A of all-cause death, all cancers, and selected cancers, stratified by type of vaccine. The unvaccinated group is the reference category for all analyses.

	BNT162b2		mRNA-1273		ChAdOx1 nCoV-19	Mixed	
	≥1 Dose [B] HR (95% CI)	≥3 Doses [C] HR (95% CI)	≥1 Dose [B] HR (95% CI)	≥3 Doses [C] HR (95% CI)	≥1 Dose [B] HR (95% CI)	≥1 Dose [B] HR (95% CI)	≥3 Doses [C] HR (95% CI)
All-cause death	0.43 (0.41-0.46)	0.64 (0.61-0.67)	0.57 (0.52-0.62)	0.73 (0.69-0.78)	0.76 (0.57-1.01)	0.26 (0.24-0.28)	0.55 (0.52-0.58)
All cancers, [D]	1.27 (1.13-1.44)	1.10 (1.02-1.19)	1.09 (0.94-1.27)	1.06 (0.95-1.18)	2.04 (1.40-2.98)	1.16 (1.03-1.31)	1.13 (1.05-1.21)
Cancer by site [D]							
- Colon-rectum	1.24 (0.90-1.70)	1.03 (0.84-1.26)	1.15 (0.76-1.72)	1.03 (0.78-1.37)	1.11 (0.33-3.69)	1.42 (1.04-1.94)	1.29 (1.07-1.56)
- Lung	0.95 (0.70-1.28)	0.95 (0.79-1.15)	0.78 (0.52-1.18)	0.80 (0.59-1.09)	2.50 (0.97-6.45)	0.84 (0.62-1.14)	0.98 (0.81-1.19)
- Breast	1.49 (1.03-2.14)	1.27 (0.98-1.65)	1.39 (0.87-2.22)	1.40 (1.01-1.92)	3.65 (1.24-10.8)	1.59 (1.11-2.28)	1.42 (1.11-1.81)
- Uterine, body	1.76 (0.71-4.38)	1.27 (0.73-2.21)	2.38 (0.82-6.89)	1.35 (0.67-2.71)	NE	1.53 (0.61-3.81)	1.18 (0.68-2.04)
- Ovarian	1.99 (0.66-5.96)	2.12 (0.75-5.96)	1.58 (0.42-6.04)	NE	NE	1.35 (0.44-4.13)	1.70 (0.59-4.89)
- Prostate	0.91 (0.58-1.43)	0.86 (0.65-1.16)	0.86 (0.49-1.53)	0.80 (0.51-1.23)	NE	1.04 (0.68-1.59)	1.02 (0.79-1.32)
- Bladder	1.65 (1.05-2.57)	1.42 (1.06-1.92)	1.68 (0.98-2.88)	1.61 (1.14-2.29)	2.28 (0.51-10.1)	1.49 (0.96-2.34)	1.40 (1.04-1.87)
- Thyroid	1.95 (0.99-3.84)	1.20 (0.79-1.83)	1.47 (0.62-3.44)	0.72 (0.33-1.57)	NE	1.02 (0.49-2.12)	0.76 (0.48-1.21)
- Hematological, all	1.50 (1.07-2.10)	1.15 (0.94-1.42)	1.11 (0.71-1.73)	1.05 (0.78-1.42)	4.62 (1.97-10.8)	1.19 (0.84-1.69)	1.07 (0.87-1.33)

[A] Based on Cox proportional hazards models, adjusted for age, gender, infection status, diabetes, hypertension, cardiovascular or cerebrovascular disease, chronic obstructive pulmonary disease, kidney disease, and previous cancers (the latter only for all-cause death, as all people with cancer diagnoses up to December 31 2020 were excluded from the analyses on the cancer outcomes).
[B] When comparing the unvaccinated versus the subjects who received at least one dose, the follow-up started on March 30, 2021 for the unvaccinated individuals, and 180 days after the first dose for the vaccinated subjects.
[C] When comparing the unvaccinated versus the subjects who received at least three doses, the follow-up started on September 28, 2021 for the unvaccinated, and 180 days after the third dose for the vaccinated subjects. This analysis was not run for ChAdOx nCoV-19 as the sample size was too small to calculate HR.
[D] Subjects with at least one hospital admission (from the Italian SDO database of administrative discharge abstracts) during the follow-up, with the following ICD-9-CM codes in any diagnosis field: 140.xx–172.xx or 174.xx–208.xx (any cancer), 153.xx-154.xx (colon-rectum cancer), 162.xx (lung cancer), 174.xx (breast cancer), 182.xx (uterine cancer, body), 183.xx (ovarian cancer), 185.xx (prostate cancer), 188.xx (bladder cancer), 193.xx (thyroid cancer), 200.xx-208.xx (hematological cancers).
NE = not estimable due to the scarcity of observations.

EXCLI Journal 2025;24:690-707 – ISSN 1611-2156
Received: March 30, 2025, accepted: May 07, 2025, published: July 01, 2025

Table S3: Adjusted hazards ratios (95% confidence interval – CI) A of all cancers, and selected cancers. The unvaccinated group is the reference category for all analyses. Sensitivity analyses adopting a different start of follow-up: adding a minimum period of 90 days, instead of 180, from the start of the vaccination campaign (or the first or third vaccine dose) and the possible outcome.

	**≥1 Dose [B] HR (95% CI)**	**p***	**≥3 Doses [C] HR (95% CI)**	**p***
All cancers, [D]	1.17 (1.06-1.29)	0.002	1.08 (1.01-1.15)	0.018
Cancer by site [D]				
- Colon-rectum	1.21 (0.92-1.58)	0.17	1.15 (0.97-1.35)	0.10
- Lung	0.85 (0.66-1.10)	0.22	0.95 (0.81-1.12)	0.6
- Breast	1.42 (1.05-1.92)	0.021	1.24 (1.02-1.52)	0.031
- Uterine, body	1.52 (0.73-3.19)	0.27	1.37 (0.84-2.21)	0.21
- Ovarian	1.37 (0.58-3.25)	0.5	1.41 (0.68-2.92)	0.4
- Prostate	1.02 (0.70-1.47)	0.9	0.92 (0.74-1.14)	0.5
- Bladder	1.59 (1.07-2.36)	0.023	1.28 (1.01-1.63)	0.042
- Thyroid	1.71 (0.91-3.19)	0.09	1.05 (0.74-1.51)	0.8
- Hematological, all	1.24 (0.93-1.67)	0.15	1.07 (0.90-1.28)	0.4

[A] Based on Cox proportional hazards models, adjusted for age, gender, infection status, diabetes, hypertension, cardiovascular or cerebrovascular disease, chronic obstructive pulmonary disease, kidney disease, and previous cancers (the latter only for all-cause death, as all people with cancer diagnoses up to December 31 2020 were excluded from the analyses on the cancer outcomes).
[B] When comparing the unvaccinated versus the subjects who received at least one dose, the follow-up started on March 30, 2021 for the unvaccinated individuals, and 90 days after the first dose for the vaccinated subjects.
[C] When comparing the unvaccinated versus the subjects who received at least three doses, the follow-up started on September 28, 2021 for the unvaccinated, and 90 days after the third dose for the vaccinated subjects.
[D] Subjects with at least one hospital admission (from the Italian SDO database of administrative discharge abstracts) during the follow-up, with the following ICD-9-CM codes in any diagnosis field: 140.xx–172.xx or 174.xx–208.xx (any cancer), 153.xx-154.xx (colon-rectum cancer), 162.xx (lung cancer), 174.xx (breast cancer), 182.xx (uterine cancer, body), 183.xx (ovarian cancer), 185.xx (prostate cancer), 188.xx (bladder cancer), 193.xx (thyroid cancer), 200.xx-208.xx (hematological cancers).
* Wald test for the significance of the association between vaccination with “≥1 Dose” or “≥3 Doses” and the selected outcomes.

EXCLI Journal 2025;24:690-707 – ISSN 1611-2156
Received: March 30, 2025, accepted: May 07, 2025, published: July 01, 2025

Table S4: Adjusted hazards ratios (95% confidence interval – CI) A of all-cause death, all cancers, and selected cancers. The unvaccinated group is the reference category for all analyses. Sensitivity analyses adopting a different start of follow-up: adding a minimum period of 365 days, instead of 180, from the start of the vaccination campaign (or the first or third vaccine dose) and the possible outcome.

	**≥1 Dose [B] HR (95% CI)**	**p***	**≥3 Doses [C] HR (95% CI)**	**p***
All cancers, [D]	1.13 (0.99-1.30)	0.08	0.90 (0.83-0.98)	0.017
Cancer by site [D]				
- Colon-rectum	1.03 (0.73-1.45)	0.9	0.95 (0.77-1.19)	0.7
- Lung	0.78 (0.56-1.10)	0.16	0.69 (0.56-0.85)	<0.001
- Breast	1.63 (1.03-2.60)	0.038	1.04 (0.79-1.38)	0.8
- Uterine, body	1.45 (0.51-4.12)	0.5	0.70 (0.39-1.25)	0.23
- Ovarian	3.54 (0.57-26.4)	0.22	NE	-
- Prostate	0.79 (0.48-1.28)	0.3	0.73 (0.54-0.98)	0.034
- Bladder	1.82 (1.05-3.15)	0.033	1.07 (0.77-1.49)	0.7
- Thyroid	0.87 (0.42-1.81)	0.7	0.80 (0.53-1.21)	0.29
- Hematological, all	1.22 (0.81-1.83)	0.3	0.89 (0.70-1.13)	0.3

[A] Based on Cox proportional hazards models, adjusted for gender, age, diabetes, hypertension, cardiovascular or cerebrovascular disease, chronic obstructive pulmonary disease, kidney disease, infection status, and previous cancers (the latter only for all-cause death, as all people with cancer diagnoses up to December 31 2020 were excluded from the analyses on the cancer outcomes).
[B] When comparing the unvaccinated versus the subjects who received at least one dose, the follow-up started on January 1, 2022 for the unvaccinated individuals, and 365 days after the first dose for the vaccinated subjects.
[C] When comparing the unvaccinated versus the subjects who received at least three doses, the follow-up started on July 1, 2022 for the unvaccinated, and 365 days after the third dose for the vaccinated subjects.
[D] Subjects with at least one hospital admission (from the Italian SDO database of administrative discharge abstracts) during the follow-up, with the following ICD-9-CM codes in any diagnosis field: 140.xx–172.xx or 174.xx–208.xx (any cancer), 153.xx-154.xx (colon-rectum cancer), 162.xx (lung cancer), 174.xx (breast cancer), 182.xx (uterine cancer, body), 183.xx (ovarian cancer), 185.xx (prostate cancer), 188.xx (bladder cancer), 193.xx (thyroid cancer), 200.xx-208.xx (hematological cancers).
* Wald test for the significance of the association between vaccination with "≥1 Dose" or "≥3 Doses" and the selected outcomes.
NE = not estimable due to the scarcity of observations.
